# Microarray analyses of otospheres derived from the cochlea in the inner ear identify putative transcription factors that regulate the characteristics of otospheres

**DOI:** 10.1371/journal.pone.0179901

**Published:** 2017-06-29

**Authors:** Takehiro Iki, Michihiro Tanaka, Shin-ichiro Kitajiri, Tomoko Kita, Yuri Kawasaki, Akifumi Mizukoshi, Wataru Fujibuchi, Takayuki Nakagawa, Tatsutoshi Nakahata, Juichi Ito, Koichi Omori, Megumu K. Saito

**Affiliations:** 1 Department of Clinical Application, Center for iPS Cell Research and Application, Kyoto University, Kyoto, Japan; 2 Department of Otolaryngology Head and Neck Surgery, Kyoto University Graduate School of Medicine, Kyoto, Japan; 3 Information and Security Office, Center for iPS Cell Research and Application, Kyoto University, Kyoto, Japan; 4 Department of Hearing Implant Sciences, Shinshu University School of Medicine, Kyoto, Japan; 5 Department of Cell Growth and Differentiation, Center for iPS Cell Research and Application, Kyoto University, Kyoto, Japan; 6 Hearing Communication Medical Center, Shiga Medical Center Research Institute, Shiga, Japan; University Claude Bernard Lyon 1, FRANCE

## Abstract

Various tissues possess tissue-specific stem/progenitor cells, including the inner ears. Stem/progenitor cells of the inner ear can be isolated as so-called otospheres from differentiated cells using a sphere forming assay. Although recent studies have demonstrated the characteristics of otospheres to some extent, most of the features of these cells are unknown. In this report, we describe the findings of transcriptome analyses with a cDNA microarray of otospheres derived from the cochleae of the inner ears of neonatal mice in order to clarify the gene expression profile of otic stem/progenitor cells. There were common transcription factors between otospheres and embryonic stem cells, which were supposed to be due to the stemness of otospheres. In comparison with the cochlear sensory epithelium, the otospheres shared characteristics with the cochlea, although several transcription factors specific for otospheres were identified. These transcription factors are expected to be essential for maintaining the characteristics of otospheres, and appear to be candidate genes that promote the direct conversion of cells into otic stem/progenitor cells.

## Introduction

Hearing is essential for communication. Approximately 360 million people suffer from hearing impairment worldwide [1], which results in a lower quality of life for these patients. The perception of sound involves the cochlear sensory epithelium (CSE), which contains hair cells and supporting cells. Hair cells are the transducers of auditory stimuli into neural signals, and are surrounded by supporting cells [2]. Sensory hearing loss mainly occurs as a result of disorders of the hair cells [3]. The hair cells can be damaged by acoustic trauma, ototoxic drugs and/or aging.

In mammals, the capacity for proliferation and regeneration in mammalian hair cells is considered to be lost after birth [4], and sensory hearing loss is almost always permanent owing to the irreversible loss of hair cells or their associated neurons [5]. Adult avian vestibular and auditory hair cells can be newly produced and regenerated after noise or ototoxic drug damage via mechanisms of cell differentiation following supporting cell division as well as direct transdifferentiation [6–12]. A recent report showed that Wnt signaling plays the main role in avian HC regeneration [6]. However, some studies have shown that hair cells in the vestibular organs of adult mammals can occasionally be regenerated *in vitro* after certain ototoxic damage [13–15]. It has also been reported that the supporting cells from neonatal mouse cochleae retained their capacity to divide and transdifferentiate into hair cells [16]. These findings indicate the possible presence of remaining stem/progenitor cells that can give rise to hair cells in the mammalian inner ear.

However, this regeneration takes place only under specific *in vivo* conditions, and is not practically present under normal conditions, suggesting that the cochlear sensory epithelium harbors dormant stem/progenitor cells that are able to differentiate upon specific types of stimulation. Therefore, innovative cell therapies, such as those promoting the expansion, directed differentiation and transplantation of these stem cells, may provide a cure for hearing loss. Stem/progenitor cells have been proven to be harbored in the CSE via the generation of floating spheres, called otospheres, when cells dissociated from the CSE were subjected to a suspension culture [17,18].

This sphere formation assay is similar to the neurosphere assays, where multipotent and self-renewing cells can be isolated from the central nervous system in mammals [19–21]. Using this technique, the isolation of stem/progenitor cells from the vestibular and cochlear regions has been successfully performed [17,18,22–29]. Otospheres have a capacity for self-renewal, express markers of the developing inner ear, such as Sox2 and Nestin, and are capable of differentiating into a variety of cell types of the inner ear, including hair cells, supporting cells and neurons.

Recent studies have shown that the capacity of otospheres for self-renewal and multipotency are regulated by the cell cycle and Wnt or Notch signaling [30–32]. However, a comprehensive gene analysis has not yet been reported, and the detailed gene expression patterns regulating these abilities are generally unknown. Because stem/progenitor cells derived from various tissues share fundamental biological properties, it has been argued that these cells may share a subset of specific genes related to “stemness” [33,34]. These genes may also be expressed in a higher order pattern [35], even if they are not common genes known as universal markers of stem cells. Therefore, using a cDNA microarray, we compared the gene expression pattern of otospheres with that of the CSE and embryonic stem cells (ESCs) to clarify the unique transcriptional characteristics of otospheres as tissue-specific stem/progenitor cells.

## Materials and methods

### Study ethics

All experimental protocols were approved by the Animal Research Committee of the Kyoto University Graduate School of Medicine (Permit Number. Med Kyoto 13156) and performed according to the institutional guideline of Kyoto University. Sodium pentobarbital was used for euthanasia of all mice, and all efforts were made to minimize suffering. Animal care was provided by the Institute of Laboratory Animals of Kyoto University.

### Animals and cochlear dissection

**Fig 1:**
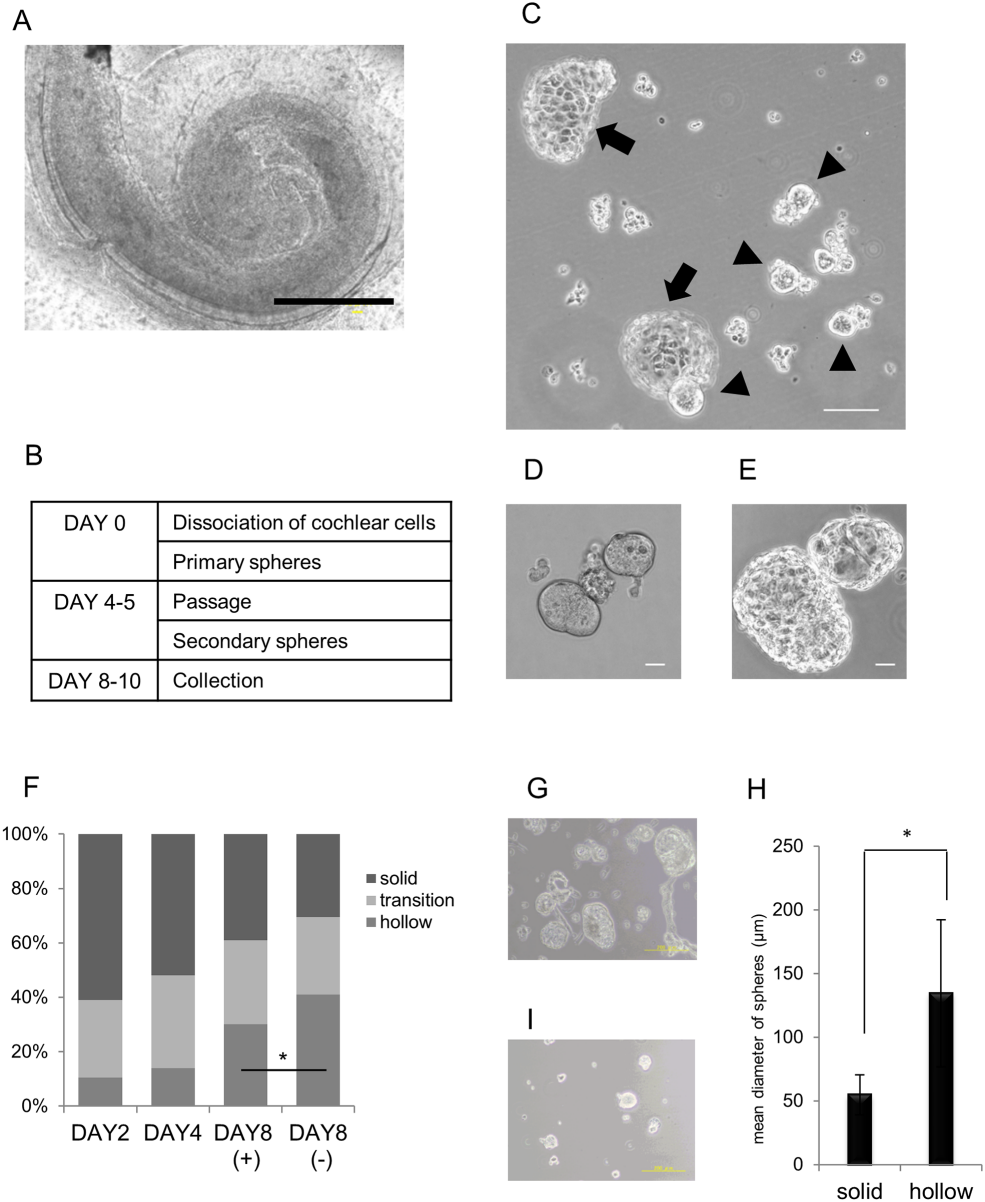
Otosphere preparation from the cochlear sensory epithelium. (A) A phase-contrast microscopic view of a neonatal mouse CSE. The scale bar represents 400 μm. (B) A time table for the culture of otospheres. (C) Otospheres formed from dissociated CSE via suspension culture for two days (arrowhead; solid type, arrow; hollow type). The scale bar represents 100 μm. (D, E) A high magnification view of the solid (D) and hollow (E) types of otospheres. The scale bar represents 10 μm. (F) The ratio of different sphere morphologies after 2, 4 and 8 days in the suspension culture. The major solid sphere population observed on DAY 2 was decreased on DAY 8. Conversely, the hollow sphere population increased on DAY 8. DAY 8 (+) or (-) means the presence or absence of the passaging of otospheres on DAY4. Fewer hollow spheres were observed in DAY 8 (+) condition (*p<0.01; Chi-square test). The ratio was obtained by the mean numbers of spheres in six plates in one experiment. (G-I) The otospheres before (G) and after (I) filtration using a cell strainer for the otospheres when they were cultured for four days after the first passage. The differences in size between the solid and hollow types of secondary otospheres are shown in (H). The hollow spheres were larger in diameter than the solid spheres (*p<0.01; Student’s *t*-test). The scale bar represents 200 μm in (G) and (I).

Newborn postnatal day 1 (P1) Institution of Cancer Research (ICR) mice were used for the study. For each experiment, the CSE was dissected from 10 mice as described previously [36] (Fig 1A). In brief, after being anesthetized and washed with 70% ethanol, the mice were decapitated and the temporal bones were dissected. Twenty cochleae were excised and the whole cochlear ducts were exposed by removing the bony capsule. The CSE was isolated by separating the spiral ligament and stria vascularis, and the isolated organs were transferred to a sterile dish containing cold Hanks’ Balanced Salt Solution (HBSS). Lgr5-EGFP-IRES-CreERT2 neonatal mice [37] was kindly provided by Dr. Tateya (Institute for Virus Research, Kyoto University).

### Tissue dissociation and otosphere culture

The tissue processing and sphere formation were performed as described previously [36]. Four batches of CSE were transferred into 100 μl of 0.125% trypsin/EDTA (Thermo Fisher Scientific) in phosphate-buffered saline (PBS) and incubated for 15 minutes at 37°C. Enzymatic digestion was then blocked by adding 100 μl of a 5 mg/ml trypsin inhibitor (Sigma-Aldrich) and a 0.6 mg/ml DNase I solution (Sigma-Aldrich) in the sphere culture medium consisting of Dulbecco's modified Eagle’s medium/F12 mixed 1:1 (DMEM/F-12, Gibco, Thermo Fisher Scientific) supplemented with N2 and B27 (Gibco, Thermo Fisher Scientific), basic fibroblast growth factor (bFGF; 10 ng/ml, R&D systems, Biotechne), mouse insulin-like growth factor-1 (IGF1; 50 ng/ml, all growth factors obtained from R&D Systems), heparan sulfate (50 ng/ml, Sigma-Aldrich) and ampicillin (50 μg/ml, Sigma-Aldrich). Epidermal growth factor (EGF) was not used because the stemness of spheres can be negatively affected by EGF treatment [38]. The concentration of bFGF was optimized by our experiment at 10 ng/ml (S1 Fig).

The tissues were carefully triturated by pipetting 40 times with plastic pipette tips (10–200 μl; Greiner). The cell suspension was filtered through a 70 μm cell strainer (BD Falcon) into the wells of a six-well low attachment plate (Greiner). The remaining cells present on the first dish were washed out with 900 μl of sphere culture medium, and this medium was passed through the cell strainer into the same well of the second plate. The same manipulation was repeated with another 900 μl of medium. The cell suspension was cultured in an incubator at 37°C with 5% CO_2_. Primary otospheres were cultured for four or five days. For expansion, the otospheres were dissociated using 0.125% trypsin/EDTA for 15 minutes at 37°C, followed by mechanical dissociation involving 40 times of repeated pipetting with plastic pipette tips (10–200 μl). Then, the cell suspension was filtered through a 100 μm cell strainer (BD Falcon), and the cells were replated in a six-well low attachment plate and cultured for four or five days to obtain secondary otospheres. A time table of the cell culture is shown in Fig 1B.

To measure the diameter of the otospheres, 10 solid and 10 hollow secondary otospheres cultured for 4 days were randomly chosen for an evaluation. Their diameters were measured using a BZ-9000 All-in-one Fluorescence Microscope (Keyence), and the mean was calculated individually for the solid/hollow otospheres. The results are expressed as the means ± standard deviation (SD). Statistical significance was determined using Student’s *t*-test. The data are representative of three biological replicates.

### Otosphere differentiation

In order to study their capacity for differentiation, secondary otospheres were dissociated with 0.125% trypsin/EDTA for 15 minutes and transferred to four-well tissue culture plates (Greiner) coated with fibronectin (10 μl/ml, Sigma-Aldrich). The cells were maintained in an incubator in differentiation medium consisting of DMEM/F-12 supplemented with N2 and B27 and ampicillin (50 μg/ml). The fate of the differentiated cells was analyzed after seven days using immunocytochemistry at 37°C with 5% CO_2_.

### Mouse ESCs

Undifferentiated ESCs (D3 and G4-2) were maintained in gelatin-coated dishes without feeder cells in Dulbecco’s modified Eagle’s medium (DMEM, Gibco, Thermo Fisher Scientific) supplemented with 10% fetal bovine serum (FBS, Gibco, Thermo Fisher Scientific), 0.1 mM 2-mercaptoethanol (Wako), 0.1 mM nonessential amino acids (Gibco), and 1,000 U/ml of LIF (Wako).

### RNA isolation

Total RNA was isolated from the CSE of P1 ICR mice, the secondary otospheres and mouse ESCs (G4-2, D3). Secondary otospheres were filtered through a 100 μm cell strainer (BD Falcon) before RNA isolation to obtain a pure batch of solid otospheres. Each specimen was soaked in Trizol (Thermo Fisher Scientific) and degraded completely. Genomic DNA was degraded by aspirating the specimen with a 22 G needle (Terumo), and was removed by collecting the supernatants after centrifugation. Chloroform and glycogen (Sigma-Aldrich) were added to the supernatants to obtain an aqueous phase, which included RNA. Total RNA was isolated from the solution via isopropanol precipitation and was washed with 70% ethanol. To prevent DNA contamination, DNase treatment of the extracted RNA was performed with TURBO^™^ DNase (Ambion). The isolated RNA was dissolved in 50 μl of RNase-free water, after which the concentration of total RNA was measured using a Nanodrop2000 (Thermo Fisher Scientific) and the quality was assessed using an Agilent 2100 Bioanalyzer (Agilent Technologies).

### Reverse transcription-polymerase chain reaction (RT-PCR)

A total of 500 ng of total RNA was reverse transcribed into cDNA using Prime Script (Takara) with oligo dT primers and random hexamer primers. The RT-PCR analysis was performed using the cDNA as a template with the primers shown in S1 Table.

### Real-time quantitative PCR (qPCR)

qPCR was performed with the ABI StepOnePlus Real-Time PCR System (Applied Biosystems) using SYBR Green fluorescence (Takara) in accordance with the manufacturer's instructions. Rplp0 was used as a reference gene to normalize the relative expression of the selected genes. The qPCR amplification was carried out using the following cycle parameters: 95°C for 30 sec, followed by 40 cycles of 95°C for 5 sec, 60°C for 20 sec and 95°C for 60 sec, and then 60°C for 30 sec. Primers were designed by ProbeFinder (Roche Life Science) (see S2 Table). For each selected gene, three biological replicates were assayed independently. Relative expression fold-changes were calculated using the 2−ΔΔCt method with the expression in ESCs as the calibrator.

### Immunocytochemistry

The specimens were fixed with 4% paraformaldehyde for 15 minutes at room temperature. After being blocked with 4% Block Ace (DS pharma Biomedical) in 0.2% Triton X-100 PBS, the specimens were incubated with primary antibodies overnight at 4°C, followed by incubation with secondary antibodies for 60 minutes at room temperature. The specimens were then counterstained with DAPI, mounted with VECTASHIELD^®^ (Vector Labs) and analyzed using an Olympus FV1000 confocal microscope. The primary antibodies used are listed in S3 Table.

### Microarray

#### Microarray data preparation

Fifty or one hundred nanograms of total RNA were used to prepare Cy3-labelled target cRNA with the Low Input Quick Amp Labeling Kit (Agilent Technologies). The target cRNAs were hybridized to SurePrint G3 Mouse GE 8×60K Microarrays (Agilent Technologies) according to the manufacturer's instructions. The microarrays were scanned, and the data were analyzed using the Bioconductor package, limma. The complete dataset for this analysis is available at the NCBI Gene Expression Omnibus using accession no. GSE93055.

#### Data processing

A three-stage data processing procedure was applied to raw signal intensities. First, a background correction was performed with the ‘backgroundCorrect’ function in the ‘limma’ package of R. Then, the quantile normalization was performed to remove the technical bias using the ‘normalizeBetweenArrays’ function in the ‘limma’ package of R. Finally, to filter out control probes and low expression probes, the 95th percentiles of the negative control probes on each array were computed, and probes that were at least 10% brighter than the negative controls on at least three arrays were stored.

#### Quality control analysis

**Fig 2:**
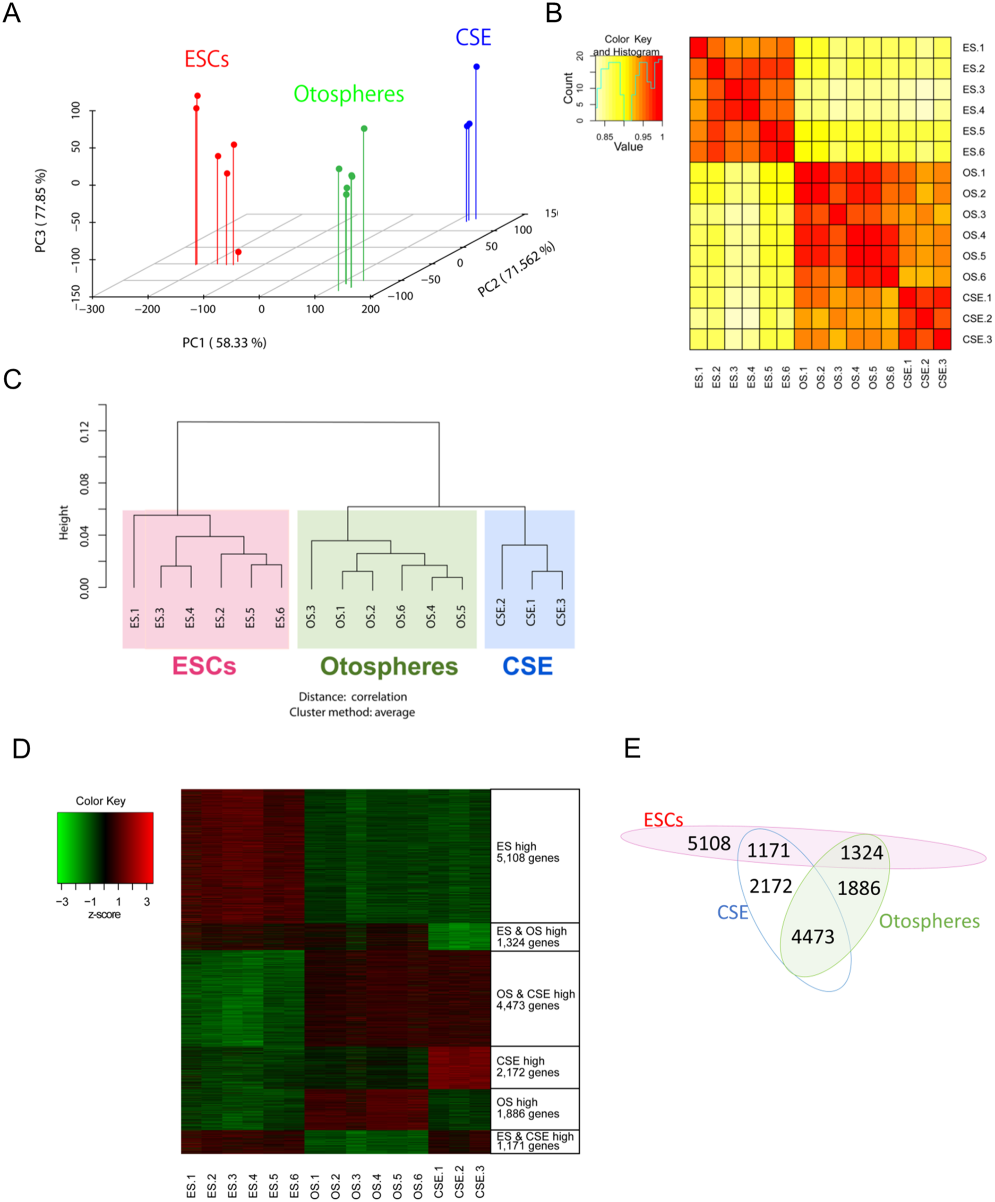
The differences observed in microarray data among the ESCs, otospheres and CSE. (A) The results of the three-dimensional principal component analysis of the three groups. The values in the brackets indicate the accumulated contribution rates. (B) The heat map displays the correlations of the gene profiles of any two samples. (C) The hierarchical clustering of samples of the ESCs, otospheres and CSE. (D) A heat map showing representative gene sets enriched among the groups according to the gene expression standardized by the z-score. (E) Venn diagram illustrating the distribution of the genes presented in (D) [49].

Expression vectors for each sample were passed to the ‘procomp’ function in the R software package, and the result were visualized as a 3D plot using to the ‘scatterplot3d’ function in the ‘scatterplot3d’ package of the R software in Fig 2A.

#### Sample-to-sample correlation analysis

The sample-to-sample correlation matrix calculated from the expression vectors for each sample was used to generate a correlation heat map using the ‘heatmap2’ in the ‘gplots’ package of the R software program without addition function al scaling, as shown in Fig 2B, and then samples were clustered according to similarity using the ‘pvclust’ function in the ‘pvclust’ package of the R software program, as shown in Fig 2C.

#### Marker gene analysis

The genes reported to be expressed specifically in the ESCs and mouse cochlea were manually retrieved from the probe sets to generate an expression matrix. The expression values were then converted to z-scores and visualized as a heat map using the ‘heatmap2’ function, and the results are shown in S2 Fig.

#### Differential expression analysis and identification of the expression pattern

To identify genes that were differentially expressed among ESCs, otospheres and CSE, statistical tests with a one-way ANOVA for each probe were performed. Multiple comparisons were then made of the false discovery rates (FDRs), and a FDR < 5% was chosen to be significant. Next, template matching was performed for classification. We generated 25 template patterns as binary vectors with a length of 3, except for (0,0,0) and (1,1,1). For each probe that was significantly differentially expressed, the Pearson’s correlation coefficient between the mean expression values for technical duplication (n = 2) and the template pattern were calculated, and the most correlated pattern was chosen as the expression pattern. A gene ontology analysis was performed using the David v6.7 software program [39].

## Results

### Otosphere generation

The cells were dissociated from the CSE of newborn mice cochleae (Fig 1A). The dissociated cells were microscopically inspected to make sure that they were completely separated into single cells, and then were cultured under floating conditions (Fig 1B). Sphere formation was found the next day (Fig 1C). As in previous reports [27,28] we classified otospheres into three subtypes based on the microscopic view: solid, transitional and hollow (Fig 1D and 1E). The solid spheres were the smallest among the three types and were found to be perfectly round with a smooth surface, while the hollow spheres were the largest, with a highly transparent thin wall. The surface of the hollow spheres displayed a polygon shape. Transitional spheres displayed an intermediate appearance between the solid and hollow types.

Collecting only solid and transitional spheres was important for the microarray analysis, because hollow spheres lose their multipotency when they differentiate into CSE components [27]. In the methods of Heller’s group, approximately 40% of spheres remained as a solid shape over 8 days of culture (Fig 1F and 1G). We therefore subcultured the otospheres to exclude the presence of contaminating cells and concentrate the stem/progenitor population. As a result, the passage of spheres by dissociating and re-aggregating cells reduced their transformation into the hollow type (Fig 1F). Primary otospheres obtained after four or five days of culture were then passaged, and secondary otospheres were further cultured for four or five days. The hollow spheres were then filtered out according to the difference in the diameter of the spheres (Fig 1H). And finally we obtained solid otospheres with more than 90% purity (Fig 1I).

### Characteristics of the solid otospheres

**Fig 3:**
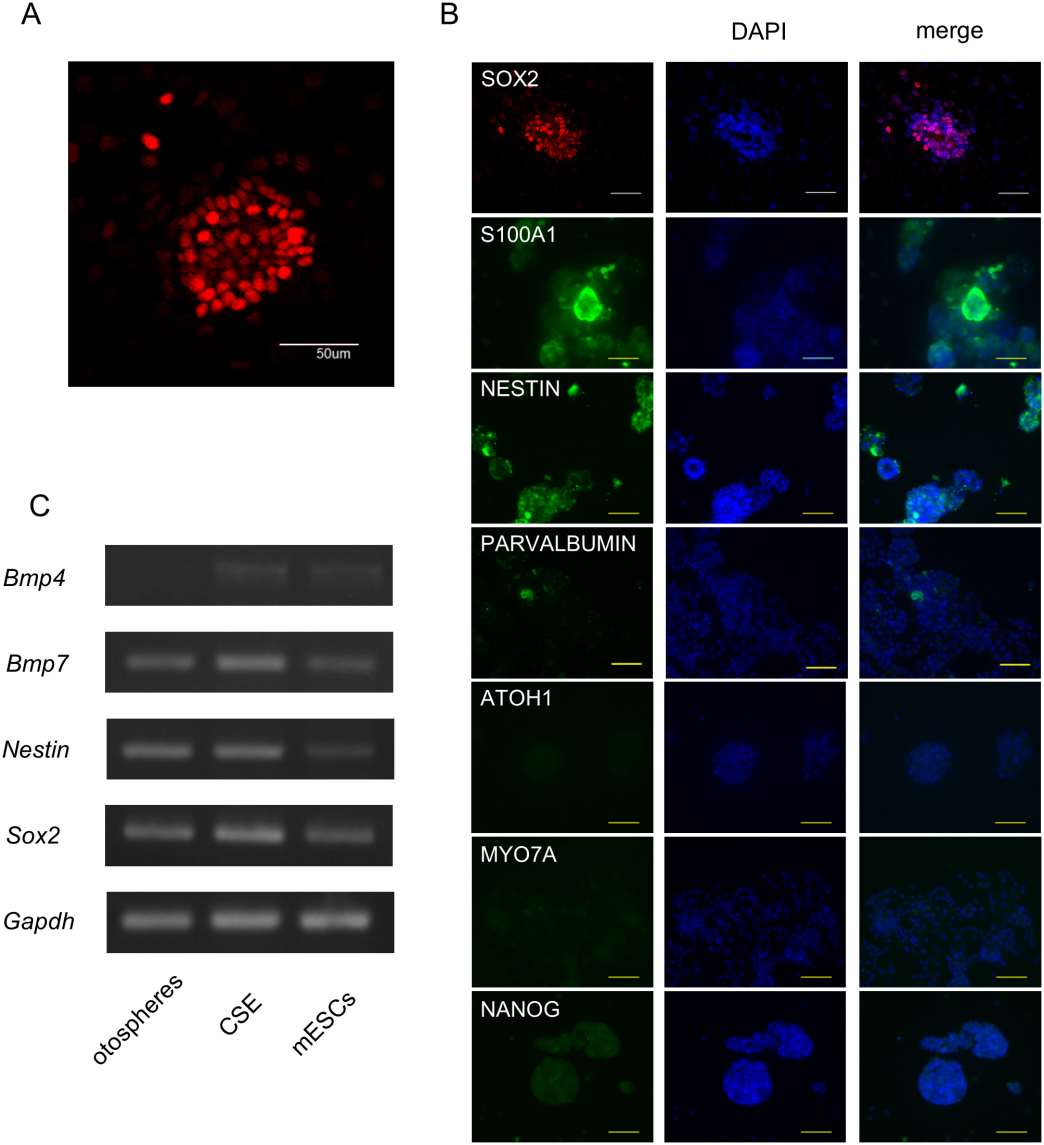
Examination of the genetic characteristics of secondary solid otospheres. (A) Representative immunostaining of solid otospheres for SOX2. The scale bar represents 50 μm. (B) Representative immunostaining of solid otospheres for proteins expressed in cochlea cells or pluripotent cells. The scale bar represents 50 μm. (C) The results of the RT-PCR analyses of several stem cell and developing cochlea markers in the secondary otospheres, neonatal cochlear sensory epithelium (CSE) and mouse ES cells (mESCs).

We next characterized the otospheres formed by our method. We first examined the expression of SOX2, which is a transcription factor involved in the development of the sensory region of the inner ear, cell fate determination and stem cell maintenance [40,41]. SOX2-positive cells were detected in all spheres, and more than 80% of cells in each sphere expressed SOX2 (Fig 3A and 3B). We also examined the expression of several genes characteristic in otospheres. The expression of stem cell markers, *Sox2* and *Nestin*, was detected in the otospheres as well as the CSE and ESCs (Fig 3C). *Bmp7*, a marker of the development of the inner ear [42], was also expressed in the otospheres, while the expression of *Bmp4* was not high. S100A1, expressed in cochlear cells [43], and NESTIN were detected in the otospheres by immunocytochemistry, but the hair cell markers PARVALBUMIN [44], ATOH1 and MYOSIN VIIA were not detected without several cells, nor was NANOG detected at all (Fig 3B), implying that otospheres have characteristics of stem/progenitor cells while retaining the expression of some major CSE-associated genes.

### Multipotency of the solid otospheres

**Fig 4:**
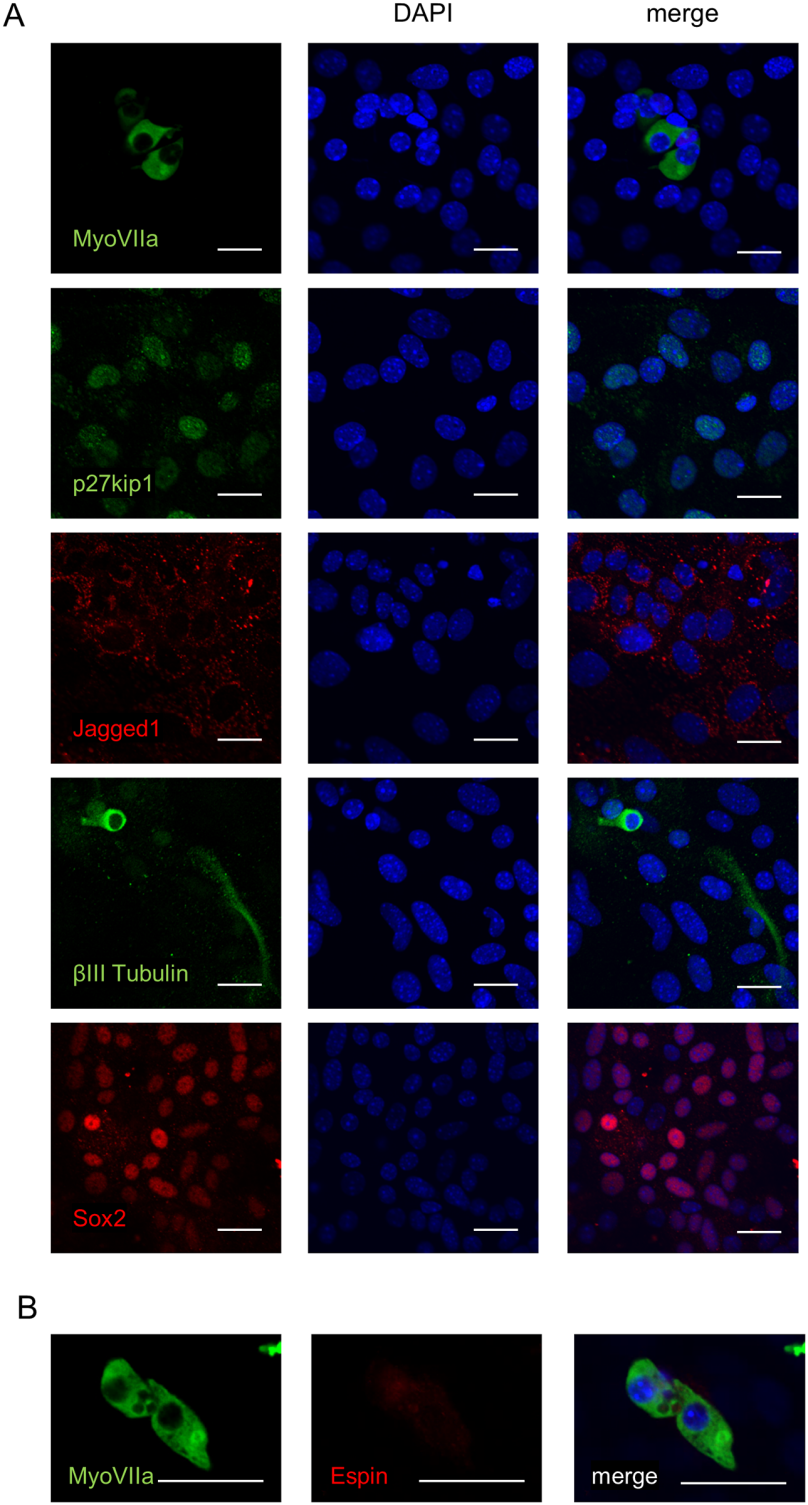
Differentiated cells from secondary solid otospheres. (A) Representative immunostaining images of otosphere-derived cells (after 7 days of differentiation) for a hair cell marker (MYOSIN VIIA), supporting cell markers (P27KIP1 and JAGGED1), neuron-specific βIII TUBULIN and SOX2. The scale bars represent 20 μm. (B) Representative immunostaining of hair cell-like cells for MYOSIN VIIA and ESPIN. The scale bars represent 20 μm.

Next, the differentiation capacity of the secondary otospheres (Fig 4A) was evaluated. Otospheres were differentiated via an adhesion culture. There were a few scattered cells expressing the hair cell marker MYOSIN VIIA [45] (5.2%±3.1%), and about 20% of the differentiated cells expressed P27KIP1 (18.3%±10.3%) and JAGGED1 (22.4%±2.7%), which are expressed in supporting cells [46,47]; the latter cells formed colonies. We confirmed that there were no cells expressing markers for both hair and supporting cells coincidently. The expression of the neuronal marker βIII TUBULIN was also found to be positive in differentiated cells (17.7%±4.2%), and SOX2-positive cells were found in 64.4%±14.3% of the observed cells. These results do not mean that these cells remained undifferentiated, because SOX2 is also expressed in supporting cells [41]. Some of the MYOSIN VIIA-positive cells were co-stained for ESPIN (Fig 4B), a marker for stereocilia [48], which are located on the apical surface of hair cells. These data indicate that the collected spheres were able to differentiate into a subset of matured cochlear cells, proving their multipotency. Therefore, these spheres were used in the subsequent cDNA microarray analyses.

### Microarray analyses of otospheres, CSE and ESCs

In order to identify genes differentially expressed between otospheres and the CSE and genes commonly expressed between otospheres and ESCs, we prepared six samples of otospheres, three samples of CSE and two samples of ESCs for the microarray. In addition to the data obtained from these samples, we used four datasets from wild-type mouse ESCs (Series GSE39765; GSM978877 and GSM978878 and Series GSE36313; GSM887832 and GSM887833) registered on the Gene Expression Omnibus (GEO). These four datasets were acquired using the same type of microarray platform (SurePrint G3 Mouse GE 8x60K) as was used in the present study.

A principal component analysis (PCA) demonstrated that each kind of cell formed individual groups that excluded the other kinds of cells (Fig 2A), and there was no significant variance in the groups of otospheres and CSE. The ESCs had variance in the third component, which may reflect clonal variance and/or the difference in the mouse strains. A heat map of the correlation between the two samples showed high correlations in the same kinds of cells, and also indicated that the otospheres had a higher correlation with the CSE than with ESCs (Fig 2B). Similar relationships were found in the hierarchical clustering analysis (Fig 2C). These results suggest that the collected otospheres had genetic similarities, and that some of the transcriptomic profile of the CSE were retained in the otospheres.

**Fig 5:**
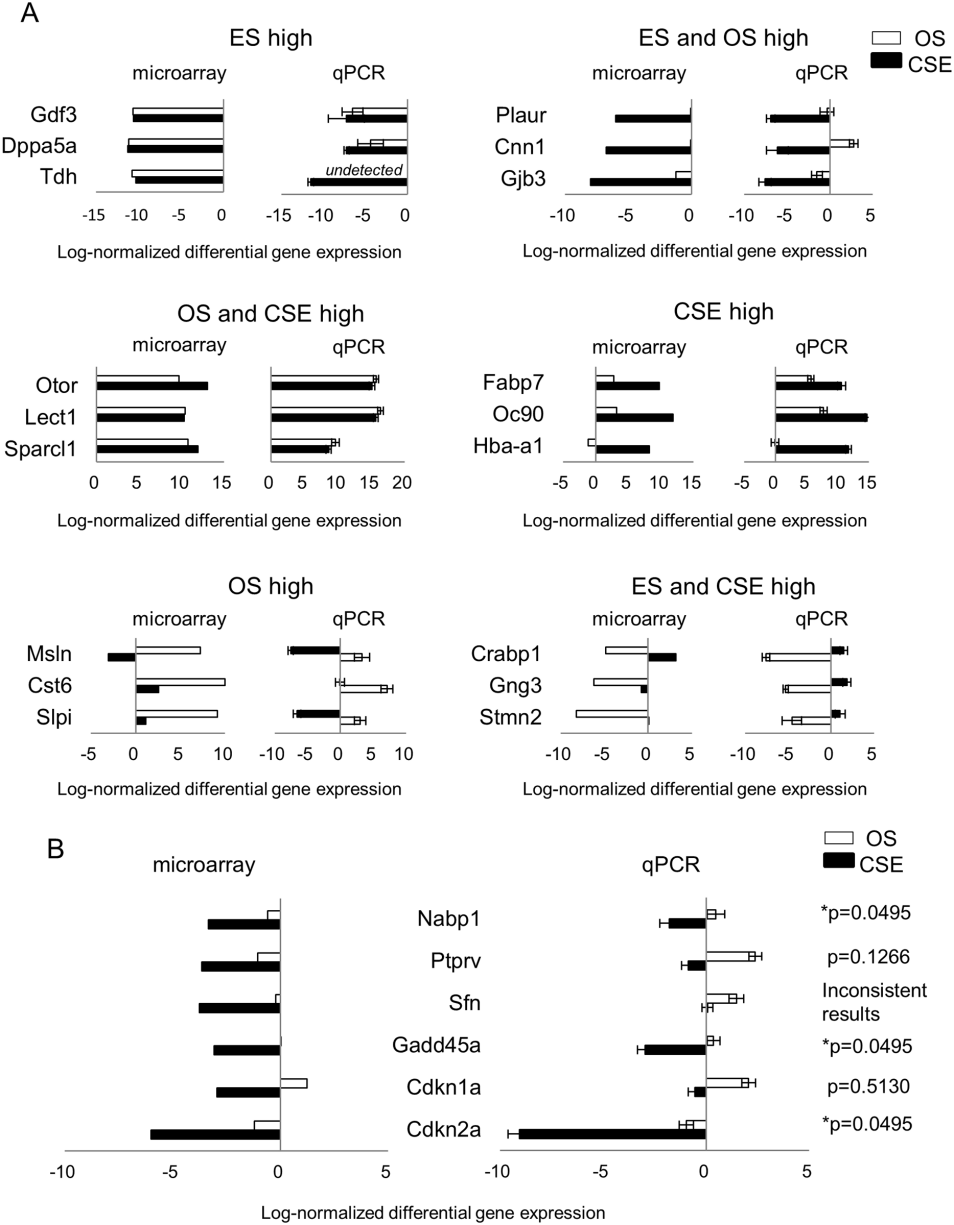
Validation of the microarray results by qPCR. (A) Mean log-normalized (log2) differential gene expression for the selected genes in Table 1A and 1B by microarray (left panel) and qPCR (right panel) derived from otospheres (green bars) and CSE (blue bars). The expression in ESC was set at 1. (B) From the microarray results, six genes in Group “ES and OS high” were identified as involved in the “regulation of cell cycle” by a GO analysis. Among them, three genes (Cdkn1a, Sfn and Ptprv) showed no siginificant difference in the gene expression between the CSE and either ES or OS. The expression in ESC was set at 1. The results are expressed as the means ± SD of three independent experiments. Statistical significance was determined using Mann-Whitney’s U test. P values of less than 0.05 are indicated with an asterisk.

To validate the microarray data, the relative gene expression of cochlear markers in otospheres and CSE compared to ESCs was examined by qPCR. The results were not inconsistent with the microarray data (Fig A in S2 Fig). We next examined the similarities and differences between each sample, focusing on general ES and cochlear markers (Fig B in S2 Fig). While the levels of ES markers were low in each sample derived from the cochlea and otospheres, the cochlear markers were not expressed ubiquitously in the otospheres. For instance, *Nestin* was expressed at lower levels in the OS3 samples than in any of the other otospheres. The levels of hair cell markers, *MyoVIIa*, *Parvalbumin*, *Atoh1* and *Pou4f3* [50], were upregulated in the OS4-6 samples, implying that OS4-6 might include cells differentiated toward hair cells. Therefore, the otospheres had some heterogeneity. Next, we identified and categorized the up- or down-regulated gene probes. There were 25,822 probes showing a significantly different expression level among the three groups (q < 0.1). Among these probes, 9,463 probes were upregulated only in ESCs (Group “ES high”), 1,883 probes were upregulated in both ESCs and otospheres (Group “ES and OS high”), 6,851 probes were upregulated in otospheres and the CSE (Group “OS and CSE high”), 3,024 probes were upregulated only in the CSE (Group “CSE high”), 2,891 probes were upregulated in otospheres only (Group “OS high”) and 1,710 probes were upregulated in the ESCs and the CSE (Group “ES and CSE high”). We then extracted the genes that satisfied the conditions of a q-value < 0.05 and a fold-change > 2 in each group. The number of genes was 5,108 in “ES high”, 1,324 in “ES and OS high”, 4,473 in “OS and CSE high”, 2,172 in “CSE high”, 1,886 in “OS high” and 1,171 in “ES and CSE high” (Fig 4D and 4E). Table 1A and 1B shows the top 10 genes with a high fold-change in each group. The differential gene expression of several select genes in Table 1A and 1B was confirmed by qPCR (Fig 5A). S4 Table shows a full and detailed list of the differentially expressed genes for each group.

**Table 1:** The 10 selected upregulated genes in each group from the microarray data.

**A**					
**ES high**		**ES and OS high**		**OS and CSE high**	
gene symbol	FC (log2)	gene symbol	FC (log2)	gene symbol	FC (log2)
Dppa5a	11.16	Gjb3	6.74	Epyc	11.22
Gdf3	10.57	Cnn1	6.70	Sparcl1	10.78
Tdh	10.31	Pmaip1	6.36	Lect1	10.36
Zfp42	10.23	Plaur	5.92	Otor	9.71
Fgf4	10.14	Krt17	5.10	Nr2f1	9.38
Pigp	10.08	Bcl3	5.01	Igfbp5	9.36
Rpl10l	10.06	Lgals3	4.88	Lum	8.98
Tex19.1	9.92	Cdkn2a	4.79	Tecta	8.51
Pou5f1	9.63	Slc7a3	4.64	Col9a1	8.46
Nanog	9.43	Hspb1	4.39	Pgm5	8.40
**B**					
**CSE high**		**OS high**		**ES and CSE high**	
gene symbol	FC (log2)	gene symbol	FC (log2)	gene symbol	FC (log2)
Hbb-b1	10.82	Wfdc18	10.44	Stmn2	8.23
Hba-a2	8.96	Lcn2	9.68	Trh	5.55
Hbb-b2	8.77	Ifi202b	8.22	Fzd10	5.43
Oc90	8.59	Slpi	8.04	Gng3	5.41
Hba-a1	8.20	Fshb	7.80	Nefh	5.22
Beta-s	7.14	Cst6	7.42	Crabp1	4.86
Fabp7	6.94	Saa3	7.40	Atp1a3	4.85
Ttr	6.90	Msln	7.24	Robo4	4.72
Serpina3a	6.50	Mmp3	7.03	Lrrc33	4.47
Cd93	6.45	Sftpd	6.88	Ramp3	4.37

### Gene ontology (GO) analyses of otospheres, CSE and ESCs

To identify the characteristics of the different cell types, we performed a GO analysis for each group. We derived enriched GO terms from the top 10% ranked lists of genes. Representative enriched biological process ontology terms, a subclass of GO terms, are described in Table 2. S5 Table shows a full list of the GO terms. Contrary to our expectations, “ES and OS high” included no GO terms associated with stemness, such as “stem cell development” (GO: 0048864). Instead of stemness, we focused on “regulation of the cell cycle” (GO: 0051726). Six genes were identified in the terms, and three genes (*Nucleic acid binding protein 1 [Nabp1]*, *Cyclin-dependent kinase Inhibitor 2A [Cdkn2a] and Growth arrest and DNA damage inducible alpha [Gadd45a]*) were significantly upregulated in both ESCs and otospheres by qPCR. (Fig 5B) “OS and CSE high” included GO terms associated with ear or neuron projection development and the cochlea, such as the sensory perception of sound and sensory perception of mechanical stimuli. “OS high” abundantly included GO terms associated with the inflammatory response, immune system and cell damage repair, which may reflect the cell damage when primary or secondary otospheres are produced. The GO term for cell adhesion was noted in “OS and CSE high”, “CSE high” and “OS high”, probably due to the formation of CSE or otospheres.

**Table 2:** The enriched gene ontologies of each group.

**ES high**			
GO term	Count	%	P value
cell cycle	68	14.0	1.79E-27
M phase	46	9.5	3.77E-25
Regulation of transcription	93	19.1	6.32E-09
Meiotic chromosome segregation	7	1.4	6.20E-08
Stem cell differentiation	8	1.6	5.49E-06
Positive regulation of cellular biosynthetic process	29	6.0	8.72E-05
Negative regulation of macromolecule metabolic process	27	5.6	1.28E-04
*In utero* embryonic development	18	3.7	1.65E-04
Anterior/posterior pattern formation	13	2.7	2.33E-04
Regulation of gene expression, epigenetic	8	1.6	0.001156
Sexual reproduction	20	4.1	0.001724
**ES and OS high**			
GO term	Count	%	P value
Negative regulation of kinase activity	5	3.6	6.60E-04
Negative regulation of cell proliferation	8	5.7	0.001846
Positive regulation of programmed cell death	8	5.7	0.003411
Programmed cell death	11	7.9	0.003825
Cytoskeleton organization	9	6.4	0.003965
Response to DNA damage stimulus	8	5.7	0.007178
Regulation of cell activation	6	4.3	0.007485
Cellular response to stress	9	6.4	0.013668
Regulation of cell cycle	6	4.3	0.026045
Posttranscriptional regulation of gene expression	5	3.6	0.028569
**OS and CSE high**			
GO term	Count	%	P value
Cell adhesion	49	11.1	3.81E-15
Ear morphogenesis	18	4.1	1.29E-12
Cell motion	35	7.9	6.08E-12
Sensory perception of sound	15	3.4	7.75E-09
Skeletal system development	26	5.9	1.32E-08
Neuron development	26	5.9	2.15E-08
Regulation of neuron differentiation	14	3.2	6.88E-07
Metanephros development	9	2.0	5.58E-05
Limb development	11	2.5	4.67E-04
Blood vessel development	15	3.4	0.001793
Inner ear receptor cell differentiation	5	1.1	0.004935
Positive regulation of transcription	21	4.7	0.008068
Locomotory behavior	13	2.9	0.010453
**CSE high**			
GO term	Count	%	P value
Neuron differentiation	16	7.5	1.30E-05
Cell adhesion	19	8.9	1.48E-05
Mechanoreceptor differentiation	6	2.8	2.94E-05
Locomotory behavior	11	5.2	1.62E-04
Wnt receptor signaling pathway	8	3.8	3.58E-04
Transmission of nerve impulse	10	4.7	4.97E-04
Cell migration	10	4.7	7.65E-04
Ion transport	16	7.5	0.00579
Forebrain development	7	3.3	0.007139
Sensory perception of sound	5	2.3	0.010504
Regulation of neurogenesis	6	2.8	0.01112
Sensory organ development	8	3.8	0.015929
Circulatory system process	5	2.3	0.026484
Transmembrane receptor protein tyrosine kinase signaling pathway	6	2.8	0.046113
**OS high**			
GO term	Count	%	P value
Inflammatory response	22	12.2	1.07E-15
Acute inflammatory response	14	7.8	3.57E-13
Taxis	6	3.3	0.003203
Multicellular organismal homeostasis	5	2.8	0.004007
Apoptosis	10	5.6	0.026505
Cell adhesion	11	6.1	0.032494
**ES and CSE high**			
GO term	Count	%	P value
Angiogenesis	5	4.3	0.004641
Pattern specification process	6	5.2	0.014583
Neuron differentiation	7	6.0	0.015627
Cytoskeleton organization	6	5.2	0.024834

### Transcription factors that regulate the characteristics of otospheres

**Fig 6:**
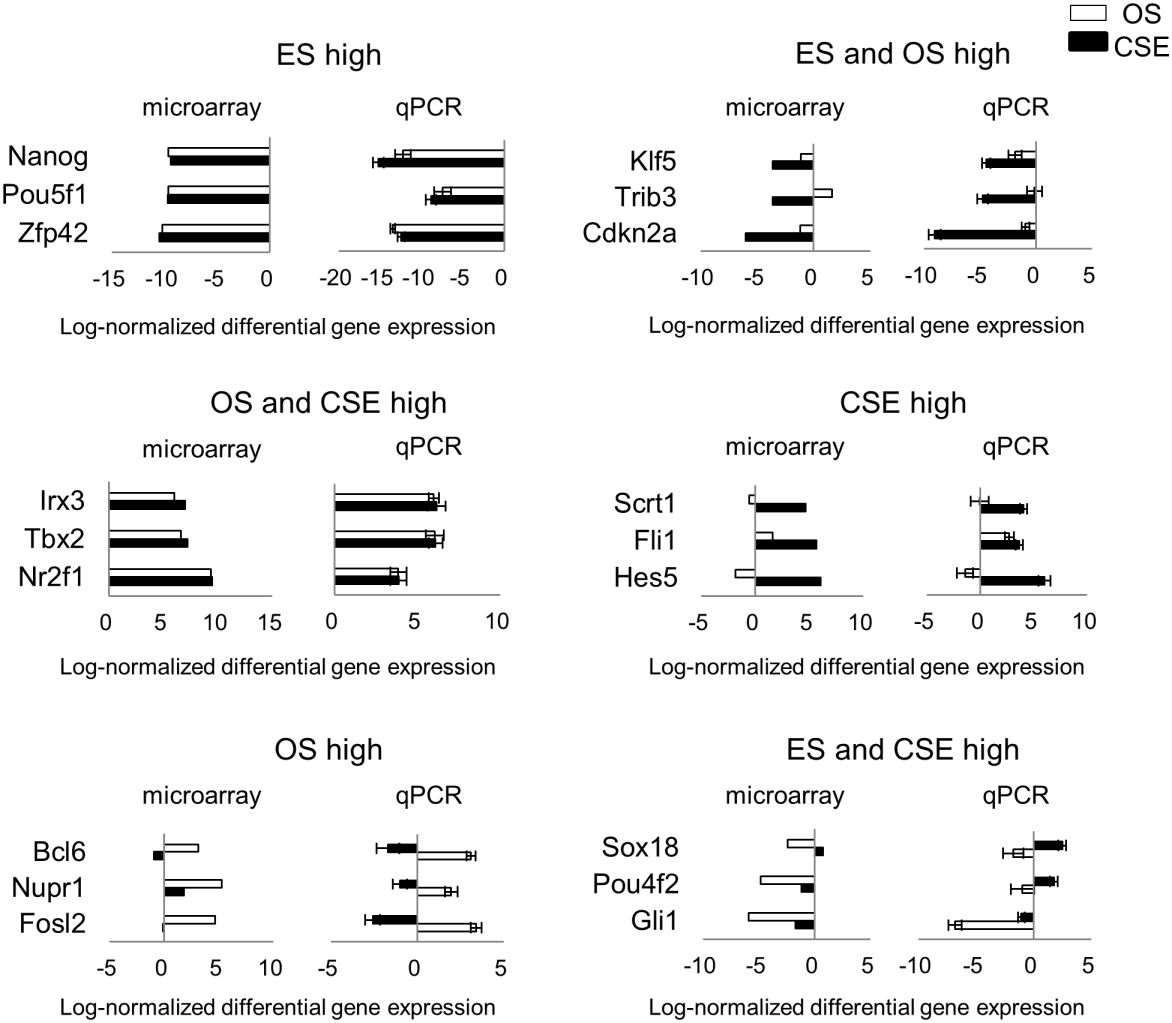
Validation of the microarray results of the transcription factors by qPCR. The mean log-normalized (log2) differential gene expression for the selected transcription factors in Table 3A and 3B by microarray (left panel) and qPCR (right panel) derived from otospheres (green bars) and CSE (blue bars). The expression in ESC was set at 1. The results are expressed as the means ± SD of three independent experiments in the right panel.

It is considered that tissue stem cells, as well as ESCs, possess a mechanism for maintaining their stemness that is fundamentally regulated by multiple genes [33,34], especially transcription factors, because these are usually located upstream of many genes in order to positively or negatively control their transcription [51]. Before identifying the transcription factors that were upregulated in each group, we narrowed the gene sets that satisfied a q-value < 0.05 and fold-change > 2 and were assigned to the GO term, “transcription, DNA-dependent” (GO: 0006351). The number of genes satisfying these conditions was 269 in “ES high”, 56 in “ES and OS high”, 181 in “OS and CSE high”, 68 in “CSE high”, 45 in “OS high” and 25 in “ES and CSE high”. We then refined the transcription factors. Table 3A and 3B shows the top 10 transcription factors with the highest fold-change in each group. The gene expression of several selected transcription factors in Table 3A and 3B by qPCR were consistent with the microarray data (Fig 6). S6 Table shows a full and detailed list of the differentially expressed transcription factors.

**Table 3:** The 10 selected upregulated transcription factors for each group.

**A**					
**ES high**		**ES and OS high**		**OS and CSE high**	
gene symbol	FC (log2)	gene symbol	FC (log2)	gene symbol	FC (log2)
Zfp42	10.23	Bcl3	5.01	Nr2f1	9.38
Pou5f1	9.63	Cdkn2a	4.79	Tbx2	6.62
Nanog	9.43	Trib3	3.64	Sox10	6.36
Zic3	8.90	Rbpms	2.57	Rarb	6.09
Dppa2	8.71	Klf5	2.51	Zfhx4	6.09
Dppa4	8.48	Sbno2	2.48	Irx3	6.04
Sall4	8.31	Baz1a	2.21	Foxg1	5.86
Zscan10	7.77	Pawr	2.18	Nfix	5.83
Tfap2c	7.61	Spib	2.15	Nfib	5.71
Gbx2	6.87	Ppp1r13l	2.09	Zbtb20	5.65
**B**					
**CSE high**		**OS high**		**ES and CSE high**	
gene symbol	FC (log2)	gene symbol	FC (log2)	gene symbol	FC (log2)
Hes5	6.08	Fosl2	4.70	Gli1	4.18
Scrt1	4.67	Nupr1	3.45	Pou4f2	3.70
Esrrg	4.22	Cebpb	3.43	Sox3	3.63
Fli1	4.08	Cebpd	3.35	Foxd3	3.56
Heyl	4.00	Bcl6	3.16	Rxrg	3.40
Pou3f2	3.71	Vgll3	3.15	Sox17	3.20
Atoh1	3.59	Atf3	3.07	Sox2	2.85
Myt1l	3.33	Fstl3	2.91	Sp5	2.56
Erbb4	3.32	Nfe2l3	2.65	Tet1	2.56
Eya2	3.25	Nfil3	2.60	Sox18	2.41

We noticed that all of the genes in “ES high” had previously been reported to be involved in pluripotency [52–61]. Other than *Hmga2*, *Cebpd* and *Bcl6*, the other transcription factors found for”ES and OS high” and “OS high” (shown in Table 3A and 3B) have not been reported to have a relationship with the cochlea [62–65].

## Expression of Lgr5 in the otospheres

**Fig 7:**
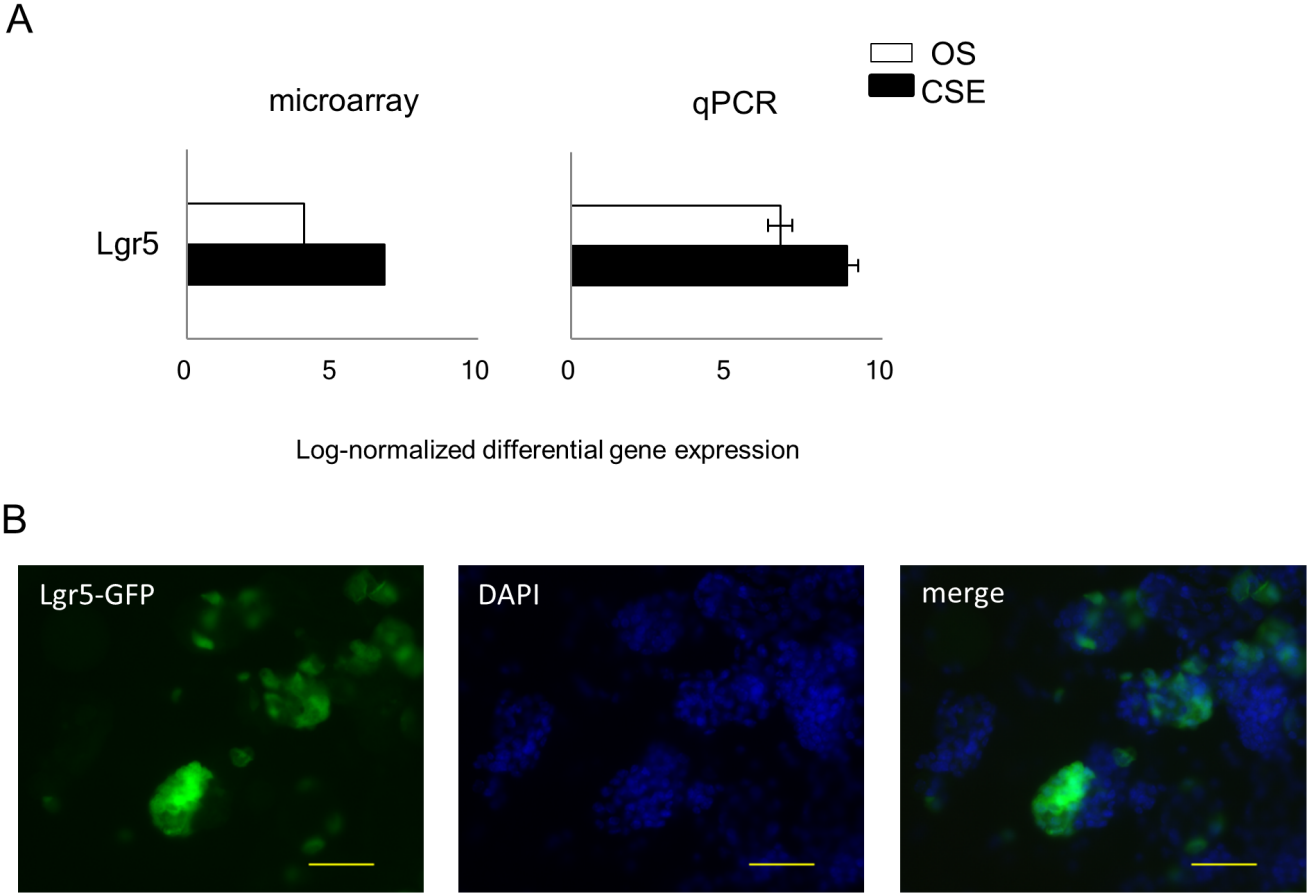
The expression of Lgr5 in the otospheres and CSE. (A) The mean differential gene expression for Lgr5 by microarray (left panel) and qPCR (right panel) derived from otospheres (green bars) and CSE (blue bars). The results are expressed as the means ± SD of three independent experiments in the right panel. (B) GFP(+) cells were detected in the otospheres derived from Lgr5-EGFP-IRES-CreERT2 neonatal mice cochlea using a fluorescence microscope. The scale bar represents 50 μm.

Lgr5 is a member of the Wnt signaling pathway and is known as a marker of adult intestinal stem cells [37]. Cochlea also has Lgr5(+) cells, and it is reported that otospheres derived from Lgr5(+) supporting cells can differentiate into hair cells at high efficiency [66]. The micoroarray analysis showed that Lgr5 was included in the “OS and CSE high” group. However, the expression of Lgr5 in the CSE was approximately eight-fold that in the otospheres, and qPCR showed similar results (Fig 7A). To examine whether or not our otospheres consist of Lgr5(+) cells, we produced otospheres using our method with cochlea of Lgr5-EGFP-IRES-CreERT2 neonatal mice [37,66]. The spheres were not formed by GFP(+) cells alone, instead including GFP(+) cells at various ratios (Fig 7B). These data confirmed the contribution of Lgr5(+) cells to our otospheres, although the otospheres may contain heterogenous cell populations.

## Discussion

Otospheres have been shown to retain the capacity for self-renewal and differentiation into the multiple cell types that form the cochlea [18,22,27,67]. This observation indicates that otospheres contain stem/progenitor cells of the cochlea and that neurospheres contain neural stem cells. Stemness of spheres is maintained by the activity of tightly regulated Sox2, which is related to the epigenetic status of the Sox2 enhancers [38]. It has been reported that the cochlea loses the majority of stem/progenitor cells during the third postnatal week [27], leading to deterioration of the sphere-forming ability of cochlear cells. Therefore, we produced and investigated otospheres using neonatal mice. Some previous studies have presented the findings of transcriptome analyses of the CSE using microarray assays [68,69]. One such study used the CSE of newborn and adult mice with the goal of detecting genes related to the maintenance or loss of otic stem cells [69]. In contrast, we tried to clearly identify the features of stem/progenitor cells in the CSE by producing and isolating otospheres from CSE samples.

The GO analysis indicated that the transcription of Nabp1, Cdkn2a and Gadd45a was upregulated in “ES and OS high” as regulators of cell cycle, which may be associated with their self-renewal. Nabp1 is essential for a variety of DNA metabolic processes, including replication, recombination and detection and repair of damage [70]. Cdkn2a encodes p16^Ink4a^ and p19^Arf^. Both proteins induce cell cycle arrest in response to stress signals [71]. The proteins encoded by Gadd45a, which is mediated by p53, also respond to stressful conditions [72]. Increased transcripts of these three genes are therefore associated with the *in vitro* subculture of otosphres. Intriguingly, Gadd45a is associated with stemness because it facilitates the reprogramming of somatic cells [73]. There were no terms related to stemness in “ES and OS high”. However, *Gjb3*, the top upregulated gene in “ES and OS high” and a member of the connexin gene family, is known to be a marker of pluripotency [74]. Interestingly, mutations in this gene can also cause non-syndromic deafness [75,76]. The ratio of transcription factors to the overall upregulated genes in “ES and OS high” was significantly larger (15.8%) than that observed in any of the other groups (5~10%). Moreover, transcription factors essential to maintaining ESCs (*Trib3*, *Klf9*) [77] were also noted to be present in “ES and OS high”. Further investigations of these transcription factors may help to elucidate the mechanisms related to the maintenance of stemness in otospheres.

Expression of cochlea-associated genes in otospheres show relative heterogeneity among experimental batches. This might be associated with the technical variability during sphere culture or selection. The other possibility for the heterogeneity may be the variability of constituent cells of otospheres. We showed that otospheres derived from CSE were not always formed by only Lgr5(+) cells. Some reports have shown that tympanic border cells, mesenchymal cells under the basilar membrane, contain slow-cycling cells that might be regarded as stem/progenitor cells [78]. Although these findings indicate the presence of putative stem/progenitor cells in the cochlea, special and temporal location of these cells have not been fully elucidated. Since otospheres are formed from entire CSE, the population of differentiating cells and stem/progenitor cells in each otospheres can vary, which may cause the heterogeneity of expression profiles.

The upregulated expression of transcription factors in ESCs samples was observed in“ES high”, “ES and OS high”, and “ES and CSE high”. While all of the genes in “ES high” shown in Table 3A are involved in pluripotency, three genes in “ES and OS high” (*Trib3*, *Klf5 and Hmga2*) [79–81] and three genes in “ES and CSE high” (*Sox2*, *Sp5 and Tet1*) [82,83] are also involved in pluripotency. On the other hand, the transcription factors included in “OS high” are specific to otospheres, and are therefore considered to be significant for maintaining the characteristics of otospheres. Recently, direct fate conversion of somatic and pluripotent cells has been successfully achieved via the introduction of several transcription factors [84–87]. Likewise, the transcription factors identified in “OS high” can be considered as candidates for deriving otic stem/progenitor cells by direct conversion, and it is expected that a cocktail of transcription factors required for the direct conversion may be identified with further experiments.

## Supporting information

S1 Fig The number of produced otospheres with various concentration of bFGF. One thousand cochlear cells dissociated were cultured in suspension for five days to obtain otospheres with various concentration of bFGF. When used bFGF at concentration of 10ng/ml, the most otospheres were obtained, however, there was no significant difference. The results are expressed as the means ± SD of three independent experiments. Statistical significance was determined using Mann-Whitney’s U test.
(TIF)

S2 Fig Gene expression of ESC markers and cochlear markers by microarray analysis and qPCR. (A) Mean differential gene expression levels for the several cochlear markers by microarray (left panel) and qPCR (right panel) derived from otospheres (green bars) and CSE (blue bars). (B) A heat map representing the similarity and divergence in the gene expression levels of the ESC markers and cochlea markers.
(TIF)

S3 Fig Uncropped gels shown in Fig 3B. A 100bp DNA ladder was used as a DNA molecular size marker in agarose gel electrophoresis. An arrow indicates non-specific bands.
(TIF)

S1 Table PCR primers. (XLSX)

S2 Table qPCR primers. (XLSX)

S3 Table Primary antibodies. (XLSX)

S4 Table A full and detailed list of the differentially expressed genes. (XLSX)

S5 Table A full list of GO terms. (XLSX)

S6 Table A full and detailed list of the differentially expressed transcription factors. (XLSX)
