# Supplementary figures and images for: Microarray analyses of otospheres derived from the cochlea in the inner ear identify putative transcription factors that regulate the characteristics of otospheres
Source: PLoS One. 2017 Jun 29;12(6):e0179901. doi: 10.1371/journal.pone.0179901 (PMC5491065; doi:10.1371/journal.pone.0179901)

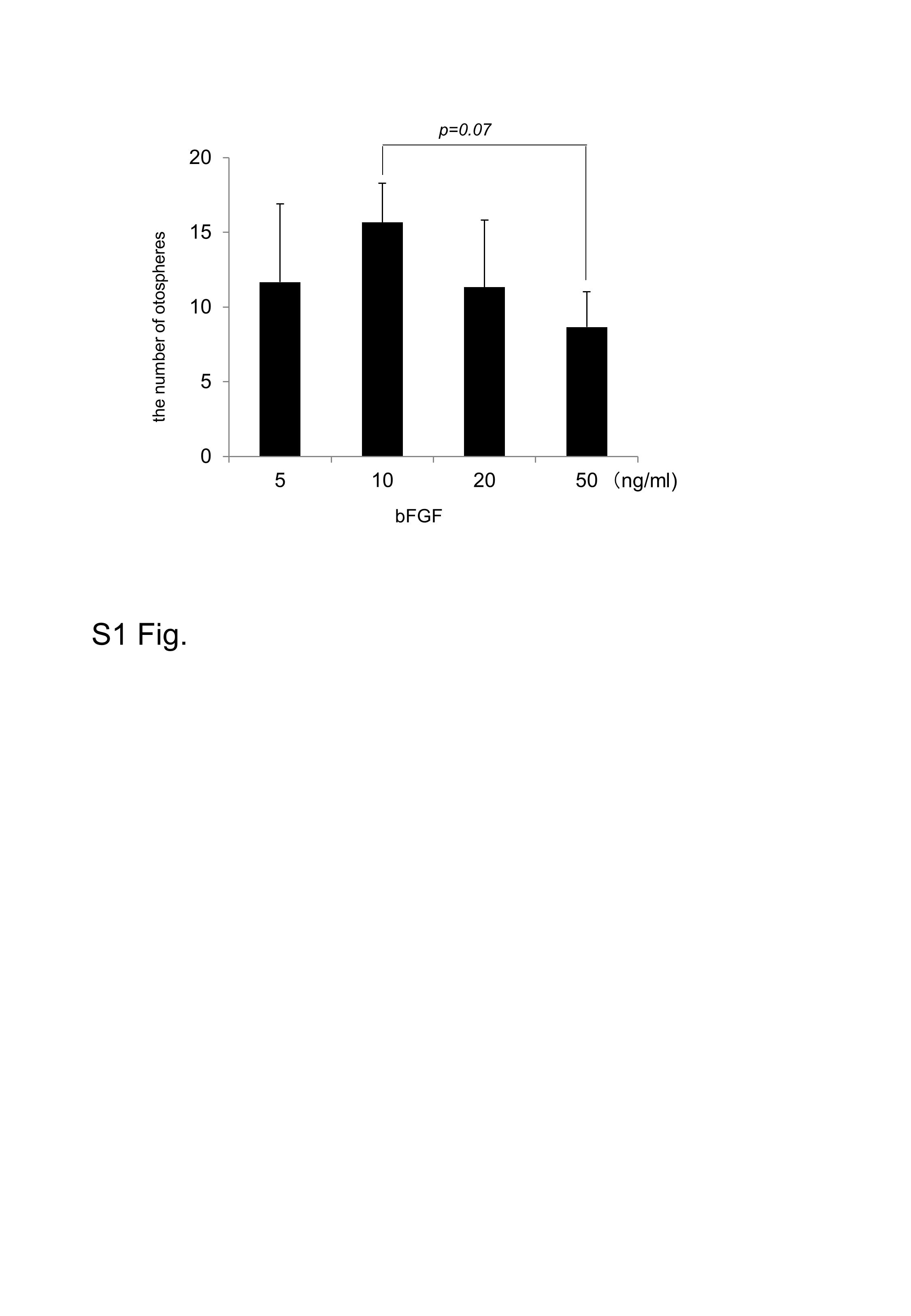

Supplement: S1 Fig — One thousand cochlear cells dissociated were cultured in suspension for five days to obtain otospheres with various concentration of bFGF. When used bFGF at concentration of 10ng/ml, the most otospheres were obtained, however, there was no significant difference. The results are expressed as the means ± SD of three independent experiments. Statistical significance was determined using Mann-Whitney’s U test. (TIF) [file pone.0179901.s001.TIF]

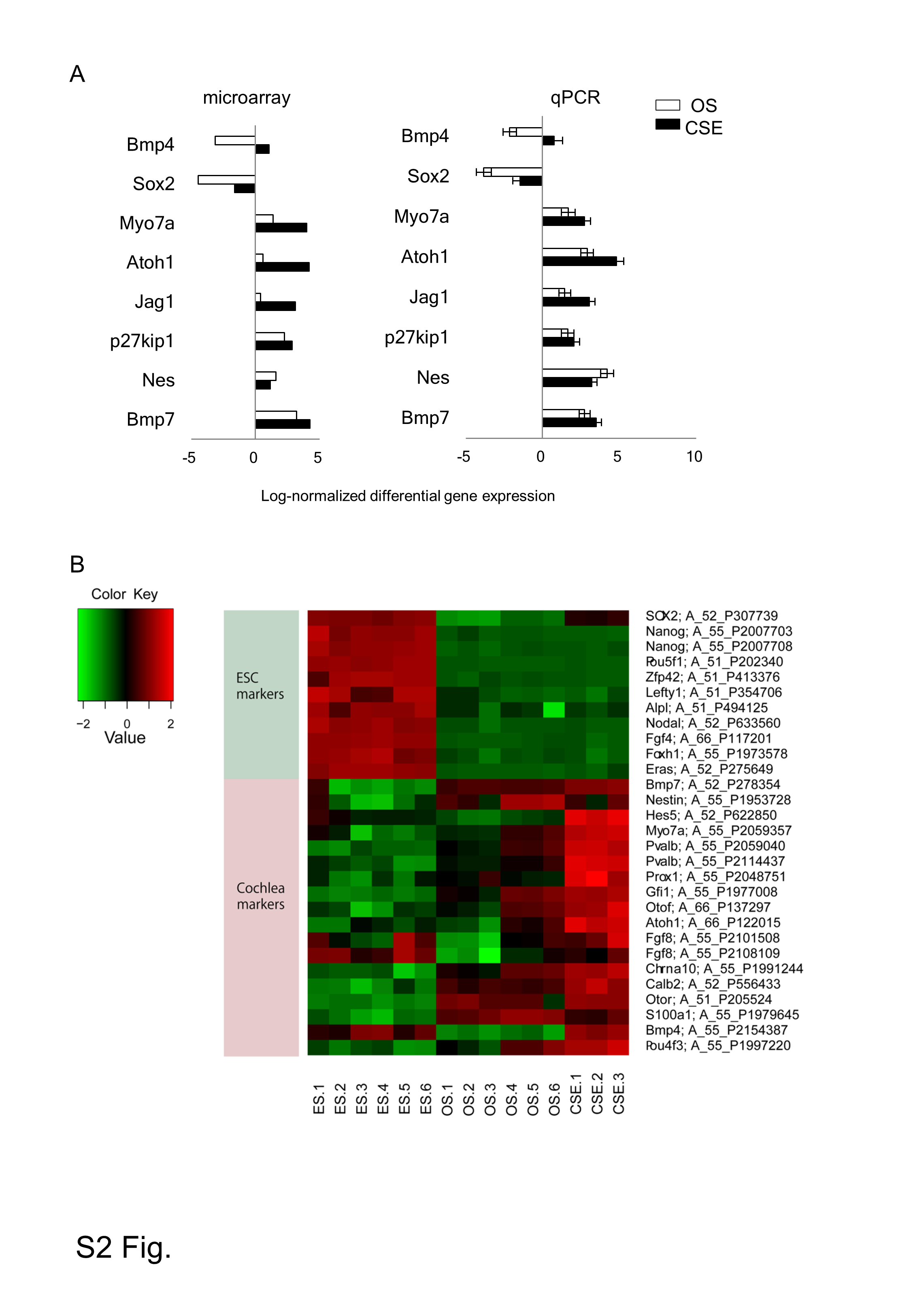

Supplement: S2 Fig — (A) Mean differential gene expression levels for the several cochlear markers by microarray (left panel) and qPCR (right panel) derived from otospheres (green bars) and CSE (blue bars). (B) A heat map representing the similarity and divergence in the gene expression levels of the ESC markers and cochlea markers. (TIF) [file pone.0179901.s002.TIF]

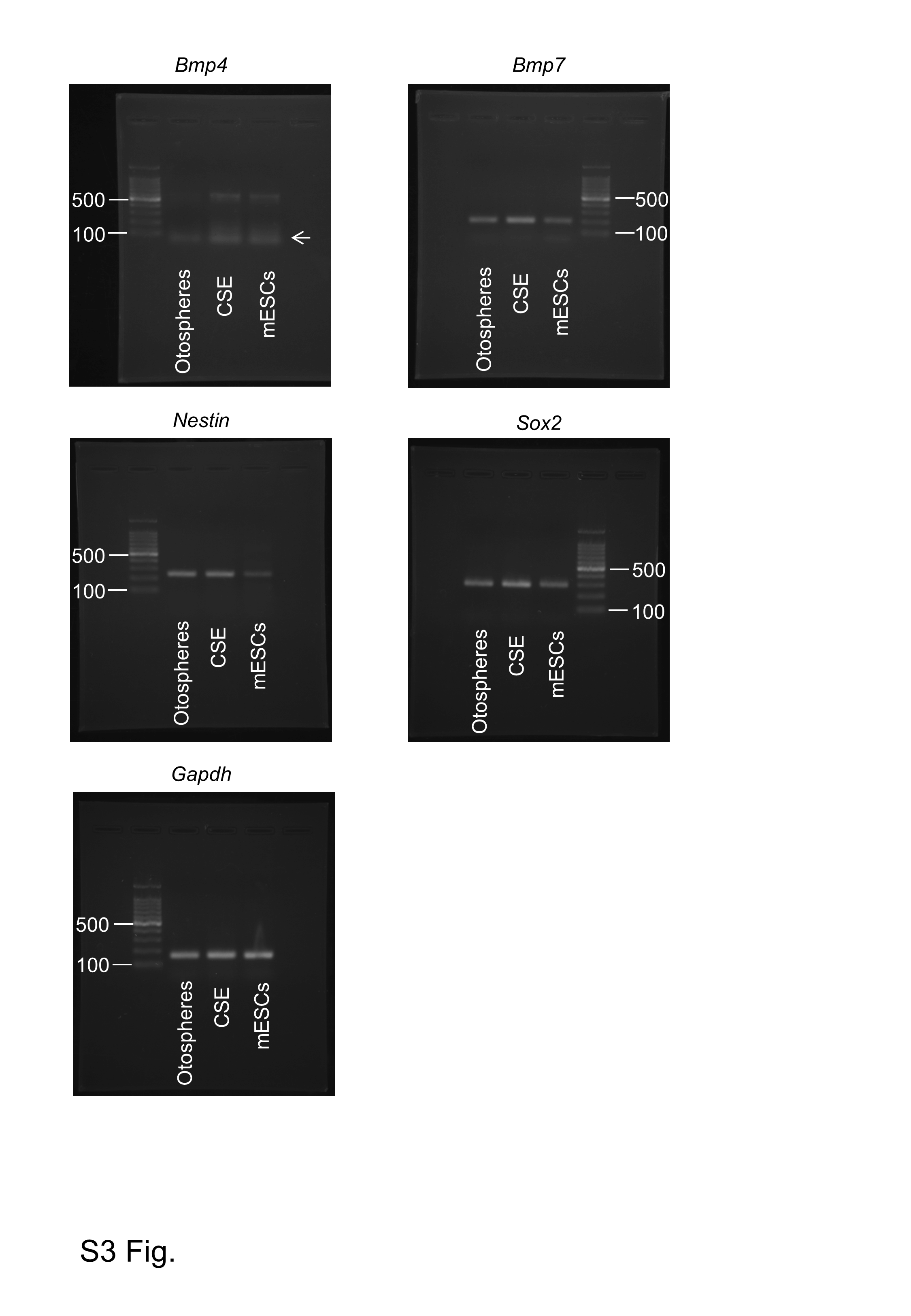

Supplement: S3 Fig — A 100bp DNA ladder was used as a DNA molecular size marker in agarose gel electrophoresis. An arrow indicates non-specific bands. (TIF) [file pone.0179901.s003.TIF]
